# How Does Digital Competence Preserve University Students’ Psychological Well-Being During the Pandemic? An Investigation From Self-Determined Theory

**DOI:** 10.3389/fpsyg.2021.652594

**Published:** 2021-04-23

**Authors:** Xinghua Wang, Ruixue Zhang, Zhuo Wang, Tiantian Li

**Affiliations:** ^1^Normal College, Qingdao University, Qingdao, China; ^2^School of Mathematics and Statistics, Qingdao University, Qingdao, China

**Keywords:** digital competence, psychological wellbeing, university students, pandemic, socioeconomic status

## Abstract

This study conceptualized digital competence in line with self-determined theory (SDT) and investigated how it alongside help-seeking and learning agency collectively preserved university students’ psychological well-being by assisting them to manage cognitive load and academic burnout, as well as increasing their engagement in online learning during the coronavirus disease 2019 (COVID-19) pandemic. Moreover, students’ socioeconomic status and demographic variables were examined. Partial least square modeling and cluster analysis were performed on the survey data collected from 695 students. The findings show that mental load and mental effort were positively related to academic burnout, which was significantly negatively associated with student engagement in online learning. Digital competence did not directly affect academic burnout, but indirectly via its counteracting effect on cognitive load. However, help-seeking and agency were not found to be significantly negatively related to cognitive load. Among the three SDT constructs, digital competence demonstrated the greatest positive influence on student engagement. In addition, female students from humanities and social sciences disciplines and lower-income families seemed to demonstrate the weakest digital competence, lowest learning agency, and least help-seeking behaviors. Consequently, they were more vulnerable to high cognitive load and academic burnout, leading to the lowest learning engagement. This study contributes to the ongoing arguments related to the psychological impact of the COVID-19 pandemic and informs the development of efficient interventions that preserve university students’ psychological well-being in online learning.

## Introduction

Digital competence is related to the knowledge, capacities, and attitudes of using digital technologies to consume, evaluate, and create learning information and to collaborate and communicate with others for learning purposes (Janssen et al., 2013; European Commission, 2019; He and Li, 2019). Developing university students’ digital competence is vital for their success in higher education (López-Meneses et al., 2020). Those with high digital competence can easily interpret and understand online learning materials and perform well in online learning (López-Meneses et al., 2020), whereas those suffering from digital deficiencies may find themselves struggling in or averse to online learning and consequently experiencing high cognitive load and academic burnout, which could eventually lead to the intention of quitting online learning (Bergdahl et al., 2020; Silamut and Petsangsri, 2020).

The unprecedented challenges caused by coronavirus disease 2019 (COVID-19) have disrupted virtually all educational institutions worldwide. As universities across nations struggle to provide continued schooling for their students, deficiencies are exposed in the large-scale remote teaching and online learning, such as complex home environments for learning, digital gap due to socioeconomic disparities, ineffective online learning systems, and inexperienced teachers (Ali, 2020; Hasan and Bao, 2020). Consequently, a series of issues arose in online learning during this pandemic, among which are high cognitive load, academic burnout, and disengagement that have been raised frequently and can impair students’ capability to learn and wreak havoc on their psychological well-being (Cao et al., 2020; Islam et al., 2020; Pohan, 2020). Under this circumstance, it calls for more digital competence on the part of students now than ever to adjust to and cope with the uncertainties.

However, there have been a limited number of empirical studies investigating students’ digital competence, particularly that of university students (Maderick et al., 2016; He and Li, 2019). Moreover, although the importance of digital competence has been widely recognized and highlighted in school settings (Hatlevik et al., 2015; López-Meneses et al., 2020), there has been limited empirical knowledge regarding how digital competence empowers students to cope with challenges in online learning and maintain psychological and emotional health, which is desperately needed for learning during this pandemic, as well as for re-entering conventional learning settings in the post-pandemic era (Cao et al., 2020; Hasan and Bao, 2020).

Nevertheless, even though digitally competent learners have potentials to perform productively and responsibly in online learning, they may not have adequate motivation for full engagement when experiencing insufficient agency and perceiving little support and help from others in online environments (Chen and Jang, 2010; Vanslambrouck et al., 2018). Therefore, to better examine how digital competence enables students to navigate through challenges in online learning, particularly during this pandemic, this study seeks to conceptualize digital competence in the framework of self-determined theory (SDT) and to examine how digital competence along with help-seeking (relatedness) and learning agency (autonomy) collectively tackles cognitive load and academic burnout and influences student engagement in online learning.

In addition, as the effects of learners’ socioeconomic status (SES) and demographic backgrounds (e.g., gender and academic disciplines) on their digital competence and responses to online learning have been much disputed in prior studies (Hatlevik et al., 2015; Mannerström et al., 2018), the present study continues to examine this topic by investigating the distribution of students according to these factors.

Specifically, this study aims to answer the following research questions:

1. How do university students’ digital competence together with their help-seeking behaviors and learning agency collectively preserve their psychological well-being by coping with cognitive load and academic burnout and enhancing their engagement in online learning?
2. How are students clustered based on their SES and demographic factors, as well as their digital competence and psychological responses to online learning?

## Theoretical Framework

### A Review of Digital Competence for Online Learning

Digital technologies are playing an increasingly important role in present days, so does digital competence, the naming of which has been controversial, some calling it Internet skills, whereas others calling it computer/digital literacy (Janssen et al., 2013; Hatlevik et al., 2015). As digital technologies are becoming smarter and user-friendlier in recent years, its naming shifts from the focus on technical skills of operating technologies in the early days to higher-order skills such as collaboration, creativity, and knowledge building (Janssen et al., 2013). Digital competence is seen as one of the crucial competences for lifelong learning by the European Union (2018), and one of the fundamental skills as writing and reading (Røkenes and Krumsvik, 2016).

Research on digital competence in the educational field has increased gradually in recent years. Most of the research was related to teacher education and sought to uncover the components forming digital competence. For instance, Alarcón et al. (2020) developed a tool to evaluate educators’ digital competence, which covered eight areas such as professional engagement, digital resources, and digital environment. Falloon (2020) presented a conceptual framework explicating a comprehensive view of teacher digital competence, which went beyond technical and literacy conceptualizations and argued for a holistic understanding that involved complex knowledge and skills needed to be productive and responsible in digital environments. Gudmundsdottir et al. (2020) examined preservice teachers’ digital competence in terms of the responsible use of digital technologies, which included topics of privacy issues, cyberbullying, and the ability to evaluate digital content.

Contrastingly, there are a few studies investigating students’ digital competence, mostly in primary and secondary schools. The topics involve the measurement of digital competence and its impacting factors. For example, Aesaert et al. (2014) developed a scale using item response theory to measure primary school students’ digital competence. The scale contained 27 items covering topics related to retrieving and processing digital information and communication with a computer. Calvani et al. (2012) investigated secondary school students’ digital competence and found that they demonstrated inadequate digital competence regarding cognitive skills and socioethical knowledge. Hatlevik et al. (2015) explored predictors of digital competence among primary school students and found that students’ motivational beliefs and family backgrounds were predictors of their digital competence levels.

However, there has been a dearth of studies examining the digital competence of university students in online learning (Maderick et al., 2016; López-Meneses et al., 2020). This may be due to the misconception about the digital competence of university students who are often assumed to be tech-savvy, growing up with the pervasive presence of digital technologies (Jena, 2015). Nonetheless, this perspective may apply to their use of technologies for entertainment or personal hobbies (Qi, 2019). When digital technologies are used intensively for educational purposes, it may be a different scenario, which entails constant cognitive and affective investment and consequently may be less appealing to the students than using technologies for recreational activities (Maderick et al., 2016; Qi, 2019).

### Digital Competence’s Importance for University Students’ Psychological Well-Being

Psychological well-being has been a frequently raised topic in this pandemic. For instance, Cao et al. (2020) investigated the psychological impact of the COVID-19 pandemic on university students and found that they experienced anxiety and worry of varying degrees. Parola et al. (2020) also found that young adults showed increased anxiety, stress, and depression during the pandemic.

The COVID-19 pandemic has changed the academic landscape and presented daunting challenges to how students take courses (Huntington-Klein and Gill, 2020). Because of the lockdowns caused by the pandemic, students are forced to study remotely at home supported by a variety of digital tools. To enable effective and productive online learning to happen, it requires the efforts of universities and teachers in the provision of digital learning resources, properly designed pedagogy, and academic support, on the one hand. On the other, it also requires the cognitive, affective, and behavioral inputs from students and particularly their digital competence to capitalize on online learning (Janssen et al., 2013; Hasan and Bao, 2020; Rasheed et al., 2020). However, students with insufficient digital competence or holding a negative perception of online learning might experience psychological distress when online learning becomes the sole means for education (Hasan and Bao, 2020).

### Conceptualizing Digital Competence in the Framework of Self-Determination Theory

Self-determined theory is a macro theory of motivation, which posits that individuals have an innate need to be self-determining or autonomous, to be competent, and to be connected to others (Deci and Ryan, 2008). SDT is considered particularly useful for examining motivation in online learning given its characteristics of flexibility and the situated and multifaceted nature of motivation (Hartnett et al., 2011; Vanslambrouck et al., 2018).

According to Deci and Ryan (2008), SDT comprises three psychological needs: autonomy (the sense of agency and volition), competence (feeling effective in attaining expected outcomes), and relatedness (interactivity and connectedness). The collective fulfillment of the three psychological needs can lead to the development of intrinsic motivation, which can keep students persistent in tackling challenges and achieving higher qualifications (Deci and Ryan, 2008; Vanslambrouck et al., 2018). Nonetheless, the deficiencies in either one of the three psychological needs may result in amotivation or external motivation. During this pandemic, when students were forced to study at home owing to the lockdowns and social distancing policies, the lack of interpersonal interactions could cause feelings of social disconnectedness and isolation, which could be magnified in virtual spaces (Evans et al., 2020; Horesh et al., 2020), consequently discouraging students from further engaging in online learning.

In this study, digital competence was used to represent “competence” according to SDT. Help-seeking behaviors were chosen as the proxy for “relatedness” because help-seeking is a social and collective process and reflects students’ sense of belongingness to a learning community based on which they cope for challenges and difficulties in online learning (Järvelä, 2011; Qayyum, 2018). Learning agency was used to represent “autonomy” as it is related to students’ self-directedness and perceived control in online learning (Kearney et al., 2015; Juhaňák et al., 2019).

### Hypotheses Development

Cognitive load refers to the amount of working memory for processing and encoding new information (Paas et al., 2003). According to Hwang et al. (2013) and Paas et al. (2003), cognitive load is assessed from two important dimensions: mental load, which is defined as the cognitive load arising from the interaction between tasks and individual characteristics; and mental effort, which is related to the cognitive capacity used for accommodating the demands imposed by learning tasks.

Admittedly, certain levels of difficulty are beneficial for learning. However, if the cognitive load is perceived high or if students deem their cognitive capacities as insufficient to achieve learning success, they tend to experience burnout, which refers to emotional exhaustion and reduced efficacy beliefs resulting from overtaxing learning tasks (Asikainen et al., 2020). Academic burnout is negatively associated with students’ psychological well-being and is closely linked to a variety of health problems, such as heavy stress, chronic fatigue, and depression (Kuittinen and Meriläinen, 2011; Salmela-Aro et al., 2019; Asikainen et al., 2020). Consequently, the students may suffer deteriorated performance and opt to give up their learning efforts (Feldon et al., 2019). This phenomenon may appear more frequently in online learning during this pandemic when students were forced to stay online for long hours to finish their coursework at home with limited resources and support available (Islam et al., 2020; Pohan, 2020). Based on the aforementioned analyses, the following hypotheses are proposed:

H1. Mental load is positively related to academic burnout.
H2. Mental effort is positively related to academic burnout.
H3. Academic burnout is negatively related to student learning engagement.

In line with SDT, individuals pursue three psychological needs (digital competence, help-seeking, and learning agency in the context of this study) in their interactions with online environments. When the three psychological needs are met and intertwined, individual students likely evaluate their behaviors as self-determined and develop intrinsic motivation (Deci and Ryan, 2008; Hartnett, 2015). Intrinsically motivated students tend to demonstrate stronger persistence in achieving expected outcomes and are less likely to be discouraged and frustrated by setbacks and challenges arising in online learning than those who are less or externally motivated (Hartnett, 2015; Vanslambrouck et al., 2018). Thus, informed by this line of reasoning, the following hypotheses are developed:

H4a-4d. Digital competence is negatively associated with mental load (H4a), mental effort (H4b), academic burnout (H4c), and learning engagement (H4d).
H5a-5d. Help-seeking is negatively associated with mental load (H5a), mental effort (H5b), academic burnout (H5c), and learning engagement (H5d).
H6a-6d. Learning agency is negatively associated with mental load (H6a), mental effort (H6b), academic burnout (H6c), and learning engagement (H6d).

In addition, help-seeking behaviors are perceived as valuable for the development of digital competence as they increase the chances of exchanging learning experiences and jointly coping with problems emerging in online environments (Røkenes and Krumsvik, 2016). Furthermore, students with high learning agency tend to perceive ownership of learning in virtual spaces and initiate actions to achieve expected learning goals, thereby gradually strengthening their digital competence of addressing challenges emerging in the learning process (Deci and Ryan, 2008; Juhaňák et al., 2019). Therefore, the following hypotheses are developed:

H7. Help-seeking is positively related to digital competence.
H8. Learning agency is positively associated with digital competence.

**FIGURE 1:**
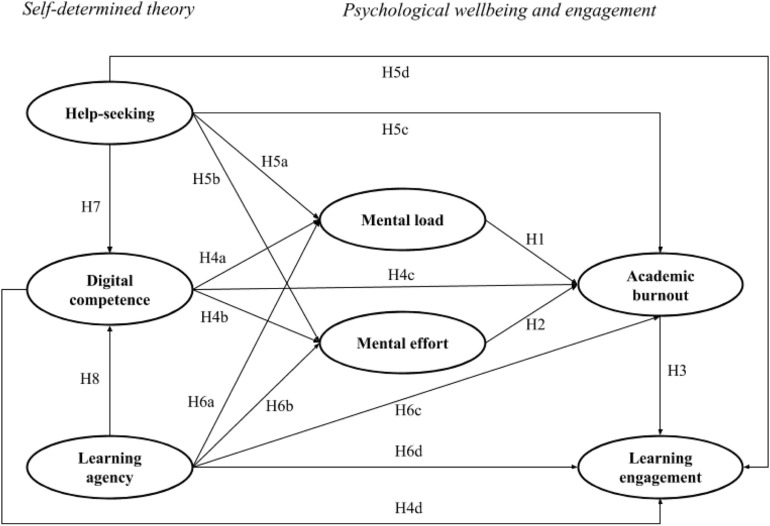
Conceptualization of digital competence in SDT and its relevance with students’ psychological well-being.

According to the hypotheses above, the theoretical research model is illustrated in Figure 1.

## Methodology

### Research Context and Participants

The sample of this study comprised students enrolled in two public universities in China. This study was conducted at the end of the semester when they had finished online courses at home due to the lockdown policy and were about to return to campus the coming semester. Before data collection, the ethical clearance of the two universities and the informed consent of all participants were obtained. The data were collected through an online survey platform using a convenience sampling approach. We approached around 900 university students with the help of their instructors and eventually obtained valid responses from 695 of them. There were 449 female students and 246 males, aged between 18 and 21 years. Among the participants, 471 students came from the disciplines of humanities and social sciences (e.g., education, psychology, and sociology), whereas 224 students from the disciplines of natural sciences and engineering (e.g., mathematics, physics, and computer sciences). To investigate the participants’ SES, they were asked to rate their family income based on a five-point scale, ranging from “0” representing the lowest-income family and “4” the highest-income family. All responses were anonymous so as to protect the participants’ privacy. Those who rated the first two points were grouped together and were labeled as “lower-income families” (*n* = 205), whereas the remaining students were labeled as “middle- and higher-income families” (*n* = 490).

### Instrumentation

The survey instrument comprised seven variables with 32 items (Appendix A), besides the items measuring students’ SES and demographic information. The items were measured using a five-point Likert scale, ranging from “1” “strongly disagree” to “5” “strongly agree,” except for help-seeking, which was scored with “1” indicating “never” and “5” “always.”

The survey was developed based on previous relevant studies. The items of digital competence were developed based on Alarcón et al. (2020); Janssen et al. (2013), and López-Meneses et al. (2020), with a Cronbach α value of 0.87. The construct of help-seeking was adapted from Qayyum (2018), with a Cronbach α value of 0.79. The items measuring learning agency were adapted from Kearney et al. (2015), with a Cronbach α value of 0.82. The items measuring mental load and mental effort were adjusted from Hwang et al. (2013), with the Cronbach α values of 0.82 and 0.85, respectively. The construct of academic burnout was adapted from Kristensen et al. (2005), and its value of Cronbach α was 0.92. Finally, the items measuring online engagement were developed based on Bergdahl et al. (2020), with a Cronbach α value of 0.94.

Considering the original instrument was in English, we followed a back-translation procedure to minimize possible differences between the English and the Chinese versions. Before administering the surveys to the participants, we invited two experts in learning sciences and two experts in psychological research to provide feedback on the survey design, based on which the survey was refined. Subsequently, the survey was tested on eight university students to examine its clarity. Items that caused confusion were reworded to convey clearer information. In addition, as this study used self-report data, Harman’s single factor test was performed to investigate possible common method bias (Podsakoff et al., 2012). We entered all variables into an exploratory factor analysis to examine the unrotated factor solution. The single largest factor explained 39.1% of the variances, which is lower than the threshold of 50%, implying that this study’s validity was not compromised by this issue.

### Analysis Methods

To validate the research model of university students’ digital competence and psychological well-being, partial least squares structural equation modeling (PLS-SEM) was used as PLS-SEM is prediction-oriented and excels at maximizing the variance explained for the dependent variables (Chin, 1998; Hair et al., 2014). To examine how the students were distributed based on their SES and demographic variables, as well as their digital competence and mental and emotional responses to online learning, a two-step cluster analysis was performed. Compared with conventional clustering techniques, the two-step cluster analysis has advantages in handling both categorical and continuous variables simultaneously, automatically determining the optimal number of clusters by comparing the values of clustering criteria across different model clustering solutions rather than arbitrary choices, and dealing with large data files (Kent et al., 2014; Benassi et al., 2020). It has been considered one of the most reliable analysis approaches for classifying individual cases into subgroups (Gelbard et al., 2007; Kent et al., 2014).

## Results

This section comprises results from PLS-SEM and the two-step cluster analysis. As for PLS-SEM, the measurement model was first examined followed by the structural model (Hair et al., 2014).

### Measurement Model

The measurement model was assessed through item reliability, convergent validity, and discriminant validity. Item reliability was attained by evaluating the item loadings with their associated latent factors, which should exceed 0.70 (Hair et al., 2014). As shown in Table 1, the loading coefficients of all items were greater than 0.70. Convergent validity was assessed through two aspects: (a) composite reliability, which should be greater than 0.70; and (b) average variance extracted (AVE), the minimum value of which should be higher than 0.50 (Fornell and Larcker, 1981; Hair et al., 2011). As indicated in Table 1, the latent constructs’ composite reliability and AVEs met the prescribed criteria. Discriminant validity was examined by comparing the square root of each latent construct’s AVE with the correlations between that and other latent constructs (Chin et al., 2003). As manifested in Table 2, all constructs’ AVEs were higher than the correlations between them and other constructs, thus substantiating the discriminant validity of the research model.

**TABLE 1:** Variable reliability, average variance extracted (AVE), and item loadings and means.

**Constructs**	**Cronbach’s α**	**Composite reliability**	**AVE**	**Indicators**	**Factor loadings**	**Mean (SD)**
Digital competence (DC)	0.87	0.90	0.61	DC1	0.71	3.75 (0.92)
				DC2	0.70	3.46 (1.05)
				DC3	0.77	3.39 (1.03)
				DC4	0.85	3.75 (0.93)
				DC5	0.85	3.44 (0.97)
				DC6	0.79	3.27 (1.01)
Help-seeking (HS)	0.79	0.87	0.62	HS1	0.77	3.06 (0.98)
				HS2	0.83	3.26 (0.99)
				HS3	0.80	3.28 (0.95)
				HS4	0.74	2.93 (0.95)
Learning agency (LA)	0.82	0.88	0.65	LA1	0.77	3.70 (0.98)
				LA2	0.88	3.58 (0.98)
				LA3	0.83	3.71 (0.92)
				LA4	0.73	3.93 (0.86)
Mental load (ML)	0.82	0.89	0.74	ML1	0.84	2.91 (1.13)
				ML2	0.87	2.49 (1.05)
				ML3	0.86	2.52 (1.05)
Mental effort (ME)	0.85	0.91	0.77	ME1	0.89	2.64 (1.13)
				ME2	0.85	3.06 (1.15)
				ME3	0.90	2.55 (1.11)
Academic burnout (ABN)	0.92	0.94	0.71	ABN1	0.76	2.92 (1.18)
				ABN2	0.90	2.42 (1.12)
				ABN3	0.89	1.99 (0.97)
				ABN4	0.79	2.41 (1.15)
				ABN5	0.88	1.89 (1.01)
				ABN6	0.81	1.91 (0.98)
Learning engagement (ENG)	0.94	0.95	0.75	ENG1	0.87	3.52 (0.99)
				ENG2	0.82	3.65 (0.94)
				ENG3	0.86	2.90 (1.02)
				ENG4	0.89	3.09 (1.05)
				ENG5	0.90	2.91 (1.02)
				ENG6	0.88	3.15 (1.00)
				ENG7	0.83	3.02 (1.14)

**TABLE 2:** Correlations between different constructs and the square root of their AVEs.

**Constructs**	**1**	**2**	**3**	**4**	**5**	**6**	**7**
1. Digital competence	**0.78**						
2. Help-seeking	0.52	**0.79**					
3. Learning agency	0.66	0.40	**0.80**				
4. Mental load	−0.32	−0.24	−0.26	**0.86**			
5. Mental effort	−0.37	−0.24	−0.29	0.71	**0.88**		
6. Academic burnout	−0.41	−0.27	−0.42	0.56	0.65	**0.84**	
7. Learning engagement	0.67	0.50	0.59	−0.37	−0.50	−0.58	**0.86**

*The bold values in the diagonal row are the square roots of the AVE of the variables.*

In addition, Table 2 shows that student engagement in online learning was significantly negatively correlated with their mental load (*r* = −0.37, *p* < 0.00, CI = −0.45 to −0.27), mental effort (*r* = −0.50, *p* < 0.00, CI = −0.57 to −0.41), and academic burnout (*r* = −0.58, *p* < 0.00, CI = −0.64 to −0.49). Moreover, significant negative correlations were also observed between the three components of SDT (digital competence, help-seeking, and learning agency) and mental load, mental effort, and academic burnout, with the correlation coefficients ranging from *r* = −0.24 (*p* < 0.00, CI = −0.28 to −0.11) to *r* = −0.42 (*p* < 0.00, CI = −0.47 to −0.32), and digital competence demonstrating the most salient negative correlations with them. All these correlation outcomes highlighted the possible detrimental effects related to high cognitive load and academic burnout in online learning during the pandemic as well as the potential counteracting effect of digital competence on them.

### Structural Model

**FIGURE 2:**
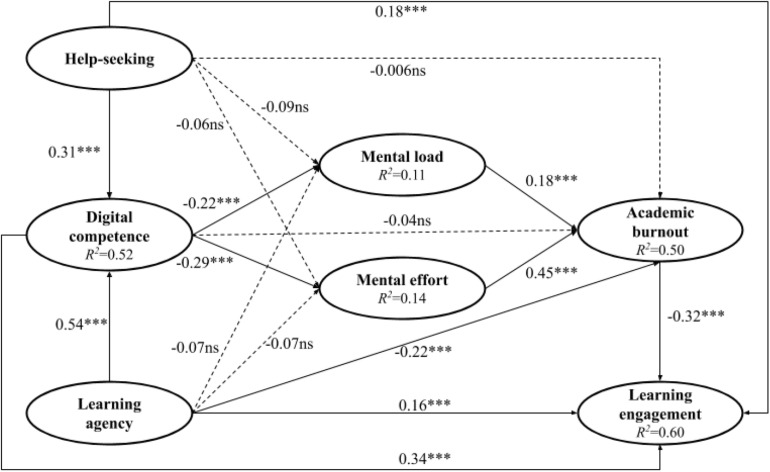
The validated research model. ^∗∗∗^*p* < 0.001. ns = non-significant. The dashed lines indicate non-significant path relationships.

To assess the structural model, we examined both the significance levels of the path coefficients in the proposed research model and the explanatory power (*R*^2^) of the endogenous constructs. Considering that parametric approaches are not suggested to evaluate the path coefficients’ significance levels as PLS-SEM does not depend on distributional assumptions, bootstrapping analysis is recommended (Sanchez, 2013; Hair et al., 2014). Table 3 presents the bootstrap results, which are illustrated in Figure 2.

**TABLE 3:** Bootstrap outcomes of the path coefficients.

**Hypotheses**		**Path coefficients**	**Standard error**	**Percentile 0.025**	**Percentile 0.975**	**Results**
H1	Mental load - > academic burnout	0.18***	0.05	0.09	0.26	Support
H2	Mental effort - > academic burnout	0.45***	0.04	0.38	0.52	Support
H3	Academic burnout - > learning engagement	−0.32***	0.04	–0.39	–0.25	Support
H4a	Digital competence - > mental load	−0.22***	0.06	–0.32	–0.12	Support
H4b	Digital competence - > mental effort	−0.29***	0.06	–0.38	–0.18	Support
H4c	Digital competence - > academic burnout	−0.04*ns*	0.04	–0.11	0.03	Not support
H4d	Digital competence - > learning engagement	0.34***	0.04	0.27	0.41	Support
H5a	Help-seeking - > mental load	−0.09*ns*	0.05	–0.20	0.001	Not support
H5b	Help-seeking - > mental effort	−0.06*ns*	0.05	–0.16	0.05	Not support
H5c	Help-seeking - > academic burnout	−0.006*ns*	0.04	–0.07	0.07	Not support
H5d	Help-seeking - > learning engagement	0.18***	0.03	0.12	0.24	Support
H6a	Learning agency - > mental load	−0.07*ns*	0.05	–0.16	0.02	Not support
H6b	Learning agency - > mental effort	−0.07*ns*	0.05	–0.18	0.03	Not support
H6c	Learning agency - > academic burnout	−0.22***	0.04	–0.30	–0.15	Support
H6d	Learning agency - > learning engagement	0.16***	0.04	0.08	0.23	Support
H7	Help-seeking - > digital competence	0.31***	0.03	0.25	0.37	Support
H8	Learning agency - > digital competence	0.54***	0.05	–0.19	–0.02	Support

***p < 0.001. ns, non-significant.

According to Table 3 and Figure 2, mental load and mental effort were positively related to students’ academic burnout in online learning, which further negatively predicted their learning engagement, thereby substantiating H1, H2, and H3, and underscoring the potentially negative consequences associated with high cognitive load and academic burnout. Significantly negative correlations were observed between digital competence and mental load and mental effort; hence, H4a and H4b were supported. Help-seeking behaviors and learning agency were not negatively related to mental load and mental effort; thus, H5a, H5b, H6a, and H6b were not substantiated. However, digital competence and help-seeking behaviors did not negatively predict academic burnout, whereas learning agency did, therefore supporting H6c but not H4c and H5c. The three components of SDT (digital competence, help-seeking behaviors, and learning agency) all positively predicted student engagement in online learning, thereby supporting H4d, H5d, and H6d. Finally, students’ help-seeking behaviors and agency in online learning were positively related to their digital competence; thus, H7 and H8 were supported.

As PLS-SEM seeks to maximize the variance explained in the endogenous constructs of the theoretical model, their *R*^2^ values are used as crucial criteria to determine the quality of the structural model (Henseler et al., 2009). Figure 2 shows that the *R*^2^ values of digital competence, mental load, mental effort, academic burnout, and learning engagement were 0.52, 0.11, 0.14, 0.50, and 0.60, respectively, suggesting medium to large effect sizes (Cohen, 1988) and thus high explanatory power of the research model. In addition, according to the global criterion of goodness-of-fit (0 < GoF < 1) that is proposed by Tenenhaus et al. (2004) to determine the overall quality of PLS-SEM, the GoF indices of 0.10, 0.25, and 0.36 indicate small, medium, and large fit, respectively. The GoF value of the PLS-SEM analysis in the present study was 0.51, implying a substantially good fit for the research model and thereby providing further supportive information about the research model.

### Two-Step Cluster Analysis

The Bayesian information criterion (BIC) was used as the clustering criterion for computing the potential numbers of clusters. Smaller BIC values suggest better models (Vrieze, 2012). Nonetheless, in the scenario where the BIC values continue to decrease while the number of clusters increases, gradually complicating the cluster model, the changes in BIC values and those in the distance measure are examined to determine the optimal cluster solution so as to balance the tradeoff between the complexity of the cluster model and the BIC values.

**FIGURE 3:**
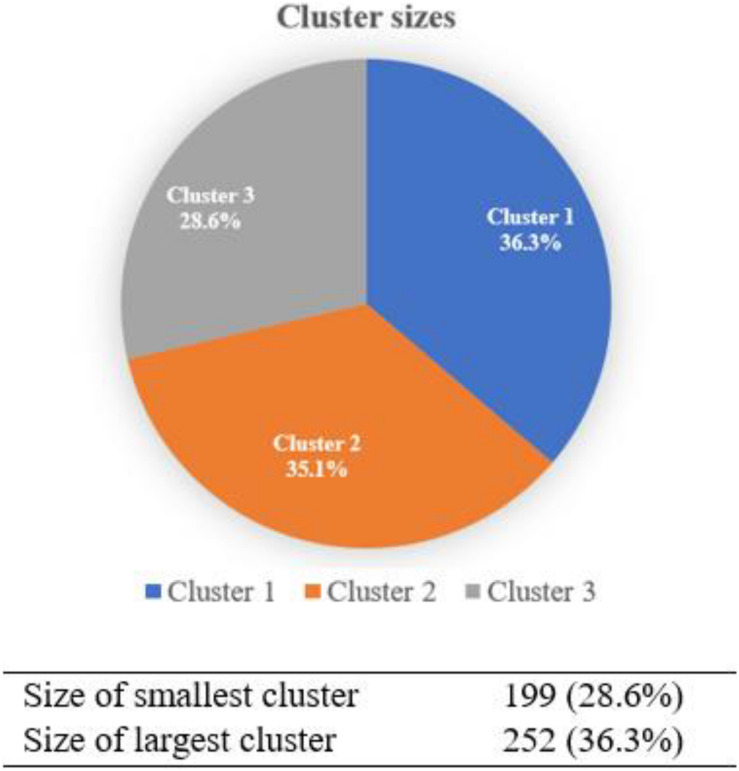
Cluster sizes for the three-cluster solution.

As shown in Table 4, the two-step cluster analysis reported a three-cluster classification as the optimal solution, with lower BIC values (5,433.58), the biggest BIC changes (0.653), and changes in distance measure (1.656). Figure 3 presents the supportive information about the three-cluster classification as the distribution of the cluster sizes was reasonable with no clusters featuring the majority of the spare parts.

**TABLE 4:** Full information of the clusters generated by the two-step cluster analysis.

**No. of clusters**	**Bayesian information criterion (BIC)**	**BIC change^a^**	**Ratio of BIC changes^b^**	**Ratio of distance measures^c^**
1	6,736.609			
2	5,948.317	–788.292	1.000	1.432
3	5,433.585	–514.732	0.653	1.656
4	5,169.327	–264.258	0.335	1.170
5	4,960.608	–208.719	0.265	1.212
6	4,809.070	–151.539	0.192	1.160
7	4,694.626	–114.444	0.145	1.005
8	4,581.267	–113.358	0.144	1.389
9	4,532.593	–48.675	0.062	1.201
10	4,511.753	–20.839	0.026	1.027
11	4,494.543	–17.210	0.022	1.086
12	4,488.059	–6.484	0.008	1.000
13	4,481.625	–6.434	0.008	1.141
14	4,490.504	8.878	–0.011	1.170
15	4,515.242	24.739	–0.031	1.138

*^*a*^The changes are from the previous number of clusters in the table. ^*b*^The ratios of changes are relative to the change for the two cluster solutions. ^*c*^The ratios of distance measures are based on the current number of clusters against the previous number of clusters.*

Table 5 displays the composition of each cluster of students examined in this study. Overall, the comparison between the three clusters implies that family income might be negatively associated with cognitive load and academic burnout. Students from lower-income families likely experience higher cognitive load and academic burnout in online learning during the pandemic. Consequently, their online engagement might be undermined.

**TABLE 5:** Summary of the three-cluster solution.

	**Cluster 1**	**Cluster 2**	**Cluster 3**
Size	36.3% (*n* = 252)	35.1% (*n* = 244)	28.6% (*n* = 199)
Input distribution	Academic disciplines NS&E (88.9%)	Academic disciplines HS (100%)	Academic disciplines HS (100%)
	Family income Middle and high income (70.6%)	Family income Middle and high income (88.1%)	Family income Lower income (51.3%)
	Gender Male (71.4%)	Gender Female (100%)	Gender Female (66.8%)
	Digital competence (mean = 3.61, SD = 0.73)	Digital competence (mean = 3.81, SD = 0.69)	Digital competence (mean = 3.02, SD = 0.65)
	Learning agency (mean = 3.73, SD = 0.73)	Learning agency (mean = 4.03, SD = 0.70)	Learning agenc y(mean = 3.36, SD = 0.71)
	Help-seeking behaviors (mean = 3.31, SD = 0.81)	Help-seeking behaviors (mean = 3.42, SD = 0.75)	Help-seeking behaviors (mean = 2.79, SD = 0.75)
	Mental load (mean = 2.64, SD = 0.98)	Mental load (mean = 2.28, SD = 0.84)	Mental load (mean = 3.07, SD = 0.75)
	Mental effort (mean = 2.74, SD = 0.99)	Mental effort (mean = 2.37, SD = 0.95)	Mental effort (mean = 3.22, SD = 0.83)
	Burnout (mean = 2.18, SD = 0.88)	Burnout (mean = 1.92, SD = 0.74)	Burnout (mean = 2.77, SD = 0.84)
	Engagement (mean = 3.32, SD = 0.85)	Engagement (mean = 3.52, SD = 0.76)	Engagement (mean = 2.58, SD = 0.76)

*NS&E, nature sciences and engineering; HS, humanities and social sciences.*

Specifically, the female students from the disciplines of humanities and social sciences and middle- and higher-income families (Cluster 2) were likely to be most motivated in the virtual classroom, demonstrating the strongest digital competence (mean = 3.81, SD = 0.69), highest learning agency (mean = 4.03, SD = 0.70), and most help-seeking behaviors (mean = 3.42, SD = 0.75). This cluster of female students tended to experience the lowest cognitive load (mental load, mean = 2.28, SD = 0.84; mental effort, mean = 2.37, SD = 0.95) and burnout (mean = 1.92, SD = 0.74) and consequently demonstrate the highest engagement (mean = 3.52, SD = 0.76) in online learning.

Contrastingly, the female students from the disciplines of humanities and social sciences and lower-income families (Cluster 3) might be the least motivated in online learning, manifesting the weakest digital competence (mean = 3.02, SD = 0.65), lowest learning agency (mean = 3.36, SD = 0.71), and least help-seeking behaviors (mean = 2.79, SD = 0.75). They were likely to experience the highest cognitive load (mental load, mean = 3.07, SD = 0.75; mental effort, mean = 3.22, SD = 0.83) and burnout (mean = 2.77, SD = 0.84) and resultantly demonstrate the lowest engagement (mean = 2.58, SD = 0.76) in online learning.

In comparison with the previous two clusters of students, the male students from the disciplines of natural sciences and engineering and middle- and higher-income families (Cluster 1) likely stand in the middle ground, demonstrating intermediate levels of digital competence, motivation, cognitive load, burnout, and learning engagement.

## Discussion

This study conceptualized digital competence in the SDT framework and investigated how it alongside help-seeking and learning agency collectively preserved university students’ psychological well-being by assisting them to manage cognitive load and academic burnout and increase their engagement in online learning, which is essential for students’ academic success in this challenging time (Ali, 2020). In addition, the roles played by students’ SES and demographic factors in this process were examined.

### Digital Competence, Help-Seeking, Learning Agency, and Challenges to Psychological Well-Being in Online Learning

As hypothesized, mental load and mental effort were positively associated with academic burnout, which further negatively affected student learning engagement. These findings are consistent with prior studies, such as those by Asikainen et al. (2020) and Chang et al. (2017), which found that high cognitive load tended to interrupt students’ learning, causing exhaustion, and frustrating experience for them and leading to disengagement and undesirable learning performance.

Contrary to the hypotheses, digital competence did not directly affect academic burnout, but indirectly through its alleviating effect on mental load and mental effort. This finding is reasonable. According to Arguel et al. (2019) and López-Meneses et al. (2020), students with higher digital competence may be in a better position to solve cognitive disequilibrium as they can make sense of digital learning materials and deal with learning requirements more effectively, thereby being more capable of addressing challenges in online learning and less likely suffering from frustrating feelings and emotional distresses.

The finding of the significant negative relationship between learning agency and academic burnout is congruent with prior studies. When students engage in online learning in a forced and unwilling way, they are more likely to have negative experiences than those who perceive control and self-directedness (Selwyn, 2016; Juhaňák et al., 2019). And when the lack of learning agency is combined with digital incompetence, the negative experience may be intensified (Selwyn, 2016).

However, help-seeking and learning agency were not significantly negatively associated with mental load and mental effort. This may be due to that mental load and effort are essentially related to digital learning materials and their instructional designs, respectively (Hwang et al., 2013). Even though seeking help when facing challenges and empowering students to exercise autonomy may contribute to easing students’ cognitive load in online learning (Qayyum, 2018; Schneider et al., 2018), it is individuals’ capabilities of processing information that largely determine the quality of interpreting and mastering the online learning materials (López-Meneses et al., 2020).

Among the three constructs used to conceptualize self-determined motivation, digital competence demonstrated the biggest influence on students’ engagement in online learning. This finding seems to be at odds with Deci and Ryan (2008) in which they argued for the element of autonomy as the core of intrinsic motivation of learning engagement. But instead, it reinforces the argument raised at the beginning of this study that students’ digital competence is greatly needed to cope with the uncertainties in online learning during this pandemic, which presents an unfamiliar context to the students (Pohan, 2020). When university courses are shifted online completely, albeit with some being unsuitable for the online instructional mode, student engagement could suffer (Huntington-Klein and Gill, 2020). The students who are more capable of capitalizing on digital resources and holding responsible attitudes toward the use of digital technologies for learning could be more prepared to tackle the challenges in uncertain times (Janssen et al., 2013).

Overall, the effect size values of digital competence, mental load, mental effort, academic burnout, and learning engagement ranged from 0.11 to 0.60 (Figure 2), implying moderate to strong effect sizes (Cohen, 1988). These results may highlight the special context caused by the pandemic when the students were forced to study online alone at home and faced with a variety of distractions and uncertainties (Cao et al., 2020). Under this context, online learning became the only means for schooling. Nonetheless, long-hour exposure to online learning could incur heavy cognitive load (Pohan, 2020). Consequently, the students tended to easily feel exhausted and overwhelmed, leading to decreased engagement and even the intention of dropping out (Bergdahl et al., 2020; Hasan and Bao, 2020). However, digital competence may have the potential to break this negative chain of reactions as it can help free the working memory of the mind to process digital learning resources effectively, thereby alleviating cognitive load that is related to learning through digital technologies (Sweller, 2005, 2020). The moderate to strong effect sizes of the constructs examined in this study also substantiated the suitability of the SDT theory that conceptualized digital competence as one of the potential ways that maintain students’ psychological well-being as well as learning engagement.

### SES and Demographic Variables and Their Relationships With Digital Competence and Psychological Responses to Online Learning

Concerning SES, the findings of the relationships between family income and students’ digital competence and psychological responses are largely congruent with prior studies on digital competence (e.g., Hatlevik et al., 2015) and recent studies about the impact of the COVID-19 pandemic on university students’ health. For instance, Cao et al. (2020) found that students from higher SES were likely to experience lower psychological distresses. This may be because students from lower-income families often have limited access to digital resources and suffer from economic pressure. Consequently, they may face more obstacles and difficulties in online learning during this pandemic, thereby more likely risking to suffer higher levels of frustration, anxiety, and decreased efficacy than their counterparts from higher-income families (Hasan and Bao, 2020).

In addition, female students from lower-income families as indicated in Cluster 3 (Table 5) were more likely to experience high cognitive load and burnout. This finding is different from Cao et al. (2020), which indicated that female and male students experienced similar stresses and negative emotions in online settings. However, it corroborates the findings of Horesh et al. (2020) that women may be more vulnerable to psychological and emotional stresses than men during this pandemic. Further studies are needed to disentangle this controversy.

### Contributions and Implications

This study has the following contributions. First, the findings of this study expand our knowledge of the role of digital competence in students’ online learning, especially during the COVID-19 pandemic. Prior studies mostly focused on its measurement and its role in academic performance (e.g., Castaño-Muñoz et al., 2017; López-Meneses et al., 2020), little attention has been given to its potentials in preserving students’ mental and emotional health, which is vital to university students during the pandemic and in the post-pandemic era when the students return to normal schooling (Hasan and Bao, 2020). The conceptualization of digital competence in SDT and relating it to challenges of students’ psychological well-being in online learning provide a new perspective of understanding digital competence.

Second, this study contributes further evidence to the debates over the effect of students’ SES on their learning in online settings. Different from previous studies that argued for the effect of individuals’ SES on digital disparities (Calvani et al., 2012), the present study shows that SES may be related to not only individuals’ competence in maneuvering the digital learning resources, but also their psychological responses while working on the resources.

Third, this study also contributes to the ongoing arguments related to the psychological impacts of the COVID-19 pandemic and informs the development of efficient interventions that preserve university students’ psychological well-being (Parola et al., 2020). Psychological interventions serve as a conventional option. However, we may also think outside of the box and design interventions from a different perspective by tracing back to the source of mental and emotional problems related to online learning. As such, academic interventions that improve university students’ digital competence while prompting their agency and encouraging interpersonal communications in online learning may be a potentially effective alternative.

In addition, this study carries implications for practice in the following ways. First, the learner clusters identified in this study can inform the development of targeted, rather than one-size-fits-all, strategies and interventions that assist learners of specific population groups to adapt to online learning while maintaining their psychological well-being. To respond to the call for equal access to opportunities promised by online learning for the underrepresented population (Littenberg-Tobias and Reich, 2020), universities should increase support to female students from lower-income families so as to enhance their digital competence and learning agency, as well as to encourage their help-seeking behaviors. Such measures are promising to put them in a better position to cope with challenges in online learning, to decrease their cognitive load and academic burnout, and to strengthen their online engagement.

Second, students’ digital competence can be improved in a variety of ways. For instance, universities ensure that faculty and students have access to all the digital resources and tools that are necessary to enhance the students’ learning online and offline (Alarcón et al., 2020). They can also provide workshops helping students to manage digital learning resources effectively and assisting faculty to improve the digital competence of their students (He and Li, 2019). However, it may be a different scenario for universities from low- and middle-income countries and their students as they tend to be subject to more financial constraints than their counterparts in high-income countries (Islam et al., 2020). These universities have to make realistic and cost-saving plans that cater to their students’ needs of developing digital competence. Affordable digital resources and tools should be provided. Besides, universities and faculty can build a strong bond with their students to improve their efficacy and attitudes toward online learning (Islam et al., 2020). They can provide students with models and hands-on opportunities that scaffold the mastery of digital competence (Røkenes and Krumsvik, 2016).

Third, even though digital competence is critical for students to be successful in online learning, encouraging students’ learning agency and help-seeking behaviors is no less important. The combination of the three factors could collectively elicit intrinsic motivation from university students, which could empower them to manage cognitive load and academic burnout in online learning without the physical presence of peers and instructors, particularly during this pandemic. And fourth, the indirect effect of digital competence on academic burnout through mental load and mental effort suggests that solely enhancing students’ digital competence may not decrease students’ burnout directly. Instructors are advised to select and design learning materials and tasks of proper difficulty levels that are congruent with students’ cognitive levels (Pohan, 2020). Although being subject to the constraints of time, resources, and experience in designing well-thought-out online learning courses, they can iteratively refine their courses based on their students’ reactions and feedback, so as to reduce the extra mental effort needed for the students to master new information. In doing so, the students’ experience of academic burnout can be expected to decrease eventually.

### Limitations and Future Research

The findings of this study should be interpreted cautiously with the following limitations. First, as this study is cross-sectional, no causal relationships can be proven. Thus, future researchers are suggested to validate the findings related to digital competence’ effect on the psychological well-being of university students and the role of SES and demographic variables in longitudinal studies so as to provide stronger argumentation. Second, the data of this study were collected from two universities that have relatively modernized digital facilities to support online learning during the pandemic. As a result, the findings of this study may suffer from the issue of overrepresenting technology-rich universities. Thus, future studies are recommended to test the research findings using a more balanced sample involving different types of universities.

Despite the limitations, this study offers a starting point for more scholastic endeavors that examine how digital competence helps preserve university students’ psychological well-being and empower them for successful professional life and social participation in the future that is filled with uncertainties, challenges, and opportunities.

## Data Availability Statement

The data that support the findings of this study are available on request from the corresponding author. The data are not publicly available due to their containing information that could compromise the privacy of research participants.

## Ethics Statement

The studies involving human participants were reviewed and approved by Ethics Review Committee, QDU. The patients/participants provided their written informed consent to participate in this study.

## Author Contributions

XW designed the study, helped with data collection, and analyzed the data. RZ designed the study, analyzed the data, and wrote the manuscript. ZW and TL helped with data analysis and edited the manuscript. All authors contributed to the article and approved the submitted version.

## Conflict of Interest

The authors declare that the research was conducted in the absence of any commercial or financial relationships that could be construed as a potential conflict of interest.
