# Supplementary material for: How Does Digital Competence Preserve University Students’ Psychological Well-Being During the Pandemic? An Investigation From Self-Determined Theory
Source: Front Psychol. 2021 Apr 23;12:652594. doi: 10.3389/fpsyg.2021.652594 (PMC8102986; doi:10.3389/fpsyg.2021.652594)
Supplement: Supplementary file 1 [file Table_1.DOCX]

Supplementary Material

**Appendix A**

| **Constructs** | **Items** | **Sources** |
| --- | --- | --- |
| Digital competence | (DC1) I am good at sharing and collaborating with others effectively in digital learning environments. | Alarcón et al. (2020), Janssen et al. (2013), and López-Meneses et al. (2020) |
|  | (DC2) I am fully aware of the legal and ethical issues on the use of digital technologies. |  |
|  | (DC3) I keep abreast of the latest developments of the digital technologies used for my work. |  |
|  | (DC4) I can decide on the digital technologies that are most relevant and appropriate for my study among a variety of options. |  |
|  | (DC5) I am confident with my capability of applying digital technologies to increase my learning effectiveness and efficiency. |  |
|  | (DC6) I can find solutions to any challenges that emerge in digitally enhanced learning. |  |
| Help-seeking | (HS1) How often did you have incidental conversations with fellow students online? | Qayyum (2018) |
|  | (HS2) How often did you intentionally communicate with fellow students about specific course content, notes, assignments, resources, and administrative questions online? |  |
|  | (HS3) How often did you collaborate with fellow students on a task online? |  |
|  | (HS4) How often did you seek answers to questions online? |  |
| Learning agency | (LA1) I determined the pacing of my online learning. | Kearney et al. (2015) |
|  | (LA2) I made plans to guide my online learning. |  |
|  | (LA3) I customized online learning content based on my needs and preferences. |  |
|  | (LA4) I was active in participating in online learning. |  |
| Mental load | (ML1) It was troublesome for me to answer the questions in the online courses. | Hwang et al. (2013) |
|  | (ML2) I felt frustrated answering the questions in the online courses. |  |
|  | (ML3) I did not have enough time to answer the questions in the online courses. |  |
| Mental effort | (ME1) The way of instruction or learning content presentation in the online courses caused me a lot of mental effort. |  |
|  | (ME2) I needed to invest lots of effort in completing the learning tasks or achieving the learning objectives in the online courses. |  |
|  | (ME3) The instructional designs in the online courses were difficult to follow and understand. |  |
| Academic burnout | (ABN1) I felt overwhelmed due to online learning. | Kristensen et al. (2005) |
|  | (ABN2) I was exhausted because of online learning. |  |
|  | (ABN3) I felt every minute spent on online learning was tiring for me. |  |
|  | (ABN4) I did not have enough energy for other activities because of online learning. |  |
|  | (ABN5) Online learning was emotionally wearying. |  |
|  | (ABN6) Online learning made me feel defeated. |  |
| Learning engagement | (ENG1) I was enthusiastic about online learning. | Bergdahl et al. (2020) |
|  | (ENG2) I found online learning full of meaning and purpose. |  |
|  | (ENG3) When I was studying online, I forget everything else around me. |  |
|  | (ENG4) I got carried away when I was studying online. |  |
|  | (ENG5) I was immersed in online learning. |  |
|  | (ENG6) I felt happy when I was studying online intensively. |  |
|  | (ENG7) I continued studying online for very long periods at a time. |  |
